# Hospital and Institutional News

**Published:** 1914-10-24

**Authors:** 


### HOSPITAL AND INSTITUTIONAL NEWS.

### THE CUMBERLAND INFIRMARY, CARLISLE.

We are indebted to Dr. Lediard for a copy of the *Cumberland News* containing an article upon the report and correspondence we have published on this infirmary, which are dealt with on page 77. Dr. Lediard's letter of October 12, mentioned last week, was published in the *Lancet* of the 17th inst. That letter contains some inaccuracies, one of which shows he has not studied the last report of the infirmary. (1) The annual report for 1913 (issued in 1914) states on a slip attached to its cover "there is accommodation for 150 in-patients." (2) It was reported to us that 140 of these beds were available for occupation. We are sorry this letter did not reach us earlier, for we must refuse to believe that the tone and tenor adopted by Dr. Lediard represent the considered attitude of the governors towards Sir Henry Burdett's recent Report on this institution.

### THE HEALTH OF THE GERMAN ARMIES.

Authentic news from Germany regarding the health of the armies and the precautions taken to prevent epidemics is scarce in this country, but the official organ of the American Medical Association continues to receive from its Berlin correspondent information on these matters. In a letter dated September 5, which appears in the issue of our contemporary which reached London this week, it is stated that up to then the health of the troops had been good and that no epidemics had arisen. "To be sure," continues the letter, "a part of our troops are in a hostile country, which has not enjoyed the same hygienic advantages as our home country, and whose inhabitants are many of them carriers of the germs of infection; but the extensive foresight of the German military organisation is effective against even these obstacles. Vaccination has been strictly enforced, and, if necessary, will be carried out among the hostile inhabitants. Apparatus for examinations for typhoid, cholera, and dysentery, and vaccination material are carried with the army. . . . The wounds received in the regular course of war show a good tendency toward healing. The German first treatment, especially the German first-aid bandage, are showing good results. The bandages applied at the front remained in good position during the transportation to the rear." The army is accompanied by consulting hygienists from the ranks of the best university instructors and by a whole staff of bacteriologically trained army surgeons. The base hospitals have portable bacteriological laboratories.

### "UTTERLY INADEQUATE" HOSPITAL ACCOMMODATION.

The most remarkable question raised at the annual meeting of the National Association of Insurance Committees, which was held at Caxton Hall on the 15th inst., was the amount of hospital accommodation now available with reference to the demands made upon it. The view expressed was that hospital accommodation was utterly inadequate in most industrial centres. For this reason a resolution was passed desiring the National Health Insurance Commissioners to inquire into the present available provision for hospital treatment throughout the country, so as to secure an increase in hospital accommodation where it is required. At the meeting where this resolution was passed, Alderman G. W. Bartlett, Durham County, who presided, stated that the Association represented ninety-eight out of the 122 English Insurance Committees, and that it had federated with similar bodies in Scotland and Wales. These statements, on the Chairman's authority, lend weight to the strong criticism of the inadequacy of the hospital accommodation, especially in industrial areas, insisted on above; and in the meantime we invite the opinion of our readers on the subject. What are the views of hospital managers in industrial centres on the relation of supply and demand of hospital accommodation?

### MR. HOWARD COLLINS' DEATH.

It is with sincere regret that we announce, and our readers will learn, the death of Mr. Howard Collins, of whose work as house governor of the General Hospital, Birmingham, an account appears, with a characteristic photograph of him, on page 87 of this issue. By way of supplement to that account of his career we should like to pay a personal tribute based on the friendly co-operation and knowledge of many years. Always as ready to help as he was full of practical knowledge Mr. Collins represented the best type of those hospital administrators who have been trained in the last years of the past century. He had risen to a very responsible post by years of hard work, and by force of character, and of his ability as an administrator there can be no doubt. His training was as long and arduous as his enthusiasm for hospital work was high; and he told us no more than the truth when, in the course of conversation at the Newcastle Conference of last June, he said: "I always regard half my work as paid for, the other half as voluntary," by which he meant that it far exceeded the letter of his bond. Of course, the best work any man can do must exceed what can easily be reckoned in money; and Mr. Collins lived and worked at a time when the immense growth of hospital work had come about so quickly, that the qualities demanded in responsible administrators of the first class are hardly yet reflected in the rewards open to them. Having by ability and hard work forced his way to the front of his profession, he was naturally conscious of his powers and liked to take that foremost post to which he felt his record and capacity entitled him. But with all this no man of his position was kinder to the junior men; and we could quote more than one instance of the secretary of a small hospital who was grateful to Mr. Collins for his quick sympathy and ready advice. Indeed he was always accessible and courteous to a marked degree in a man who had so many claims upon his time and temper; and it was one of the pleasures of those who attended the conferences of the British Hospita's Association to wait for him to rise. He never failed to speak shortly, to the point, and sometimes with a good-humoured banter, to those whose opinions he felt impelled to challenge, which often enlivened the debate, brought it back if it wandered, and at the same time never gave offence. If a career so successful can have had a disappointment it may have occurred when his candidature for the post of superintendent to the Manchester Royal Infirmary was not successful; but his reputation in Birmingham could hardly have been surpassed at any other city. All who knew him will regret the loss of a fine administrator and a genial personality.

### THE POOR LAW AND THE RED CROSS.

The British Red Cross Society has written to several Boards of Guardians who have offered accommodation for the wounded stating that "no infirmary under the control of the Local Government Board is to be taken over for hospital purposes, except under direct order from the military authorities." Except in certain seaport towns, such as Bristol and Portsmouth, there has been no necessity so far to make use of Poor-Law institutions for the wounded. Many of the Belgian refugees and destitute Austrian, German, and Hungarian aliens have, however, been provided for in workhouses. Over 200 Belgians from Antwerp have been received at the Kensington Workhouse, and the Mary Place Workhouse of the same Union is to be utilised for the reception of destitute alien enemies.

### DR. KEATS AWARDED DAMAGES FOR LIBEL.

What we believe to be the last of the actions for libel which Dr. W. J. C. Keats, medical superintendent of Camberwell Infirmary, has been compelled to take with reference to certain statements issued in connection with an election to the Camberwell Board of Guardians, both by private individuals and in newspapers, has now ended like its predecessors in a verdict for the plaintiff. The cases, it will be recalled, arose out of certain allegations with reference to the punishment of child patients in the infirmary, as to the nature of which the jury failed to agree in March last (see The Hospital, March 28, p. 697). In the present instance Mr. Justice Bray, after the jury had answered a number of questions in favour of Dr. Keats, and assessed the damages at £300, allowed a stay of execution upon terms in view of an appeal. The defendants in the present case were Mrs. C. A. R. Bracey-Wright and her son, Mr. W. H. C. Bracey-Wright, whose allegations were contained in an address issued by them in support of their candidature for election to the Camberwell Board of Guardians and in an accompanying leaflet. In these the plaintiff was alleged to have flogged with "a five-tailed lash" sick and convalescent children in the infirmary. Once again we congratulate Dr. Keats upon the successful issue of his action, but the monetary damages are poor compensation for the anxiety and annoyance which the allegations must have caused. While offering him our congratulations on the result, may we also be allowed to repeat our previous conviction that evils occasioned by the condonation or practice of corporal punishment must always in the end far exceed its immediate effectiveness in securing discipline? A libel action, even if successfully brought, is certainly one of the evils of litigation which the majority of men desire to avoid; and therefore we cannot help thinking that our criticism of the Guardians in 1913, based on these grounds, for condoning corporal punishment has been fully justified by the event.

### HOSPITAL SHIPS FOR THE INDIAN TROOPS.

Having alluded to the plan for providing a hospital in a warm climate for the wounded among our Indian troops, and to the possibility of its being organised at Alexandria, it becomes interesting to learn that special hospital ships are also in requisition. According to the *Indiaman*, the Admiralty has made arrangements, with the advice of Lieut.-Col. W. A. Sykes, D.S.O., for six hospital ships for the Indian troops, apart from those which the generosity of the Indian princes may provide. Two are stated to be P. and O. vessels and four from the Union-Castle line, while all are of some 6,000 tons displacement. The accommodation on each vessel is given as 350, including 100 special swinging cots for serious cases. The arrangements were in course of completion, and, if the hospital in a warm climate was speedily available, there would have been complete provision for this vital section of our Army in France. Since writing the above, however, practical considerations have decided the authorities not to convey wounded Indian soldiers by way of Marseilles to a 500-bed hospital at Alexandria. The greater facility of conveying men to (and no doubt back again from) a spot in the north of France, or in the south of England, along with their English comrades, is one reason; while, of course, it may further be useful as showing our appreciation of India's loyal co-operation with the Empire.

### ACCOMMODATION AT NETLEY FOR INDIAN WOUNDED.

Owing to temporary accommodation for the wounded Indian troops being now provided at Netley the Indian Field Ambulance Training Corps is receiving an early opportunity for useful work. Under the command of Colonel James Baker, late I.M.S., the Corps was started to meet the wishes of numerous Indian students to participate actively in the Allied cause. Training has been in progress not only in London but at week-end camps at Eastcote, near Pinner. A detachment of thirty men has now been taken by the War Office for service at Netley, for which they left on Monday under the command of Lieut.-Colonel Sharman, I.M.S., retired. Another and smaller detachment has left for immediate service on the hospital ships set apart for Indian soldiers. Comforts for those in France have been receiving the active attention of Surgeon-General Sir Havelock Charles and Sir Trevredyn Wynne, the vice-chairman of the St. John Ambulance Association, who have visited France to study the problem of distribution on the spot.

### SUPPLIES OF VACCINE FOR THE ARMIES IN FRANCE.

The Inoculation Department of St. Mary's Hospital since the beginning of the war has supplied only a fraction less than 1,500,000 doses of vaccine to the Allied Armies. Estimated in the form of measure, this is equal to 166 gallons, and no more succinct view of the amount of work performed by this department during the war could be presented. We trust its patriotic side will be duly emphasised not only in the forthcoming annual report of St. Mary's Hospital, but also in future public statements which may be made concerning the value of the hospital's work to the nation. The staff of the inoculation department which has been engaged in this work of preparation has been under the supervision of Captain R. S. Douglas, together with Dr. Fleming, Dr. Freeman, Dr. Haden, and Dr. Mathews. In this connection we may also remind our readers of the plea for "anti-typhoid inoculation in the Army," which formed the subject of a leading article in The Hospital of September 26, wherein a middle way was carefully pointed out between "compulsion" and the lack of immunisation among Lord Kitchener's recruits.

### THE QUEEN'S CANADIAN MILITARY HOSPITAL.

In a recent issue of The Hospital we referred to the organisation of this new emergency institution, and hinted that arrangements were in progress for its installation in a building in London. As a matter of fact, the negotiations for such a site fell through, and we are now in a position to state that Sir Arthur and Lady Markham have placed Beachborough Park, near Shorncliffe, and their staff of servants at the disposition of the Canadian War Contingent Association. With the approval of the War Office, this has been equipped and organised as the Queen's Canadian Military Hospital. The donors have provided the hospital with an *x*-ray installation, and the accommodation comprises some fifty beds, which have now been occupied by wounded from Antwerp. As previously noted, the medical, surgical, and nursing arrangements are under the supervision of Sir William Osler, M.D., F.R.S., the well-known Regius Professor of Medicine at Oxford, and Mr. Donald Armour, F.R.C.S. The lady superintendent is Miss Amy MacMahon, of Toronto, and the nursing staff is composed of Canadian trained nurses. The honorary treasurer of the Association, Mr. G. C. Cassels, would welcome subscriptions at the Bank of Montreal, 47 Threadneedle Street, E.C.

### THE STAFF TROUBLE AT LAMBETH INFIRMARY.

The trouble at Lambeth Infirmary, which was referred to in these columns on September 26 and October 2, with reference to the medical staff and its shortage through the war, has again engaged the attention of the Guardians. Dr. Baly, the medical superintendent, informed the committee that Dr. P. L. J. Watkin had been called up for service with the Royal Army Medical Corps, and he obtained the services of Mr. Thomas Hugh Edey as clinical assistant at the rate of fifteen guineas per annum. His duties will be to examine patients admitted on orders signed by the medical officers, to make notes on their condition; each morning to examine and make notes on patients specified by the medical superintendent; to assist at operations; and to make pathological examinations. Dr. Baly added that these duties, although valuable, would not serve to prevent the increased responsibilities falling upon the medical staff, and he pointed out that in the event of an assistant medical officer giving up his appointment and joining either of the forces, he not only obtained more congenial work, but the Government were prepared to pay him at a much higher rate. In the case of the third and fourth assistants, by remaining, they received £160 per annum, with increased responsibility, whereas by joining the Army they would receive over £400 per year and all found. The advantage of permanent employment was of no account. He trusted, therefore, that the Guardians would be able to find some way of recognising the services they were rendering.

### SPECIAL EMOLUMENTS FOR THOSE REMAINING.

The appeal of Dr. Baly was not in vain, for the committee placed before the board a scheme for the recognition of the services of the doctors who remained in the infirmary, which was accepted by the board unanimously. The committee informed the board that in the interests of the institutions, and having regard to the excellent openings afforded in other spheres, it was extremely desirable that the remaining members of the permanent medical staff for the institutions and the out-relief districts should be induced to continue their services in their respective capacities, and, in view of this fact, coupled with the added responsibilities which will be thrown upon them consequent upon the engagement of clinical assistants, they recommended that the officers concerned—Dr. Bowen (second assistant medical officer), Dr. Miller and Dr. Keats (fourth and fifth assistant medical officers respectively)—be each awarded a gratuity of £50 in respect of the extra duties which will devolve upon them during the twelve months from August 1 last. The board decided that one-half of the gratuity shall be paid on January 1, and the remainder at the expiration of the year in the event of the officers being on these dates in the service of the board.

### MENTIONED IN DISPATCHES.

We give below the names of the principal medical men who have been included for special mention for services rendered in the field from the commencement of the campaign up to October 8, as contained in Field-Marshal Sir John French's dispatches which appeared on Monday morning. Among those thus singled out for special comment attached to the General Headquarters Staff are Major S. L. Cummins, M.D., of the Army Medical Service, who graduated at the Royal University of Ireland in 1896; Surgeon-General (temporary) T. P. Woodhouse, of the Army Medical Service, who qualified in 1880, an old St. Thomas's Hospital man; Principal Matron Miss E. M. McCarthy, the only woman mentioned in this connection; and Colonel H. S. Hickson, M.B. (possibly to be identified with Lieut.-Colonel Samuel Hickson, R.A.M.C.) Among the remaining names, about which no mistake appears possible—and there are some whose identification is a matter of doubt—are Lieut. J. L. Huggan, R.A.M.C. (killed); Lieut. Hon. H. W. Gough, R.A.M.C.; Lieut. H. J. S. Shields, R.A.M.C.; Captain M. Leckie, R.A.M.C. (attached); Captain G. A. Kempthorne, R.A.M.C. (attached); Captain H. S. Ranken, R.A.M.C. (attached); and Captain S. E. Lewis, R.A.M.C. (attached).

### MENTIONED IN DISPATCHES: ROYAL ARMY MEDICAL CORPS.

The following is the list given of officers in the Royal Army Medical Corps also mentioned in dispatches:

Birrell, Major E. T. F.
Bourdillon, Lieut. L. G.
Butler, Major S. G.
Caddell, Capt. E. D.
Cowey, Major R. V.
Dolbey, Lieut. R. V.
Ensor, Major H., D.S.O.
Fielding, Major T. E.
Foster, Major R. L. V.
Goodwin, Major T. H. J. C., D.S.O.
Grech, Major J.
Hairsine, Lieut. C. (Special Reserve).
Helm, Lieut. C.
Hinge, Major H. A.
Hopkins, Lieut. H. L. (Civil Surgeon).
Howells, Lieut. W. M.
Lathbury, Capt. E. B.
Leckie, Capt. M.
Lloyd, Major O. W.
McEntire, Capt. J. T.
Mitchell, Lieut.-Col. L. A.
Morgan, Lieut.-Col. J. C.
Murphy, Capt. J. F. (Special Reserve).
Nimmo, Capt. W. M. (attached 1st Battalion, L. North Lancs Regt.).
O'Brien-Butler, Capt. C. P. (attached 5th Lancers).
Osburn, Capt. A. C.
Preston, Lieut. R. A.
Profeit, Major C. W.
Sampson, Capt. F. C.
Stewart, Capt. H.
Ware, Capt. G. W. W.
Wyler, Lieut. (Civil Surgeon).

To this list we cannot forbear to add the name, mentioned among the N.C.O.s, of Corporal F. J. Chatting, for it would seem that he must be the "Corporal Chatting" mentioned in last week's The Hospital in the article entitled "With a British Field Ambulance in August: by an Army Medical Officer at the Front," as "a man whom I noticed particularly as he helped me with some frightened men in the street," and "whom I had observed to be braver than most." If the same man were not intended it would be a curious coincidence, and, in the absence of evidence to the contrary, our readers will like to think that the high praise given to him by The Hospital's contributor last week has also, as the list of those mentioned in dispatches seems to show, received official confirmation.

### HOSPITALS AND INSURANCE IN 1915.

Within twelve months, it is said, the actuaries will have reported on the working of the National Insurance Act. Twenty thousand additional hospital beds will be required for the treatment of insured persons as in-patients beyond the number available in the voluntary hospitals to-day. It is certain, therefore, that the whole question of hospital provision will have to be considered on its merits, and those hospital managers who cannot show that their management is efficient, up to date, and adequate to support the number of beds under their control must expect to have a rude awakening. We anticipate inferior hospitals when reported upon may be superseded by others which will be adequate to meet the scientific as well as the medical, surgical, and just claims of the poorer members of the population residing in a few county and other towns where the hospital provision is demonstrably insufficient in quality and quantity. We should welcome a great co-operative movement in support of the best of the voluntary hospitals to embrace the whole country.

### EFFECT OF EXODUS FROM EAST COAST ON HOSPITAL WORK.

Two well-known ladies, both governors of the East Suffolk and Ipswich Hospital, have set an example in real practical help of the right sort at the right time which others similarly situated throughout the country might do well to emulate. Mary Duchess of Hamilton and the Marchioness of Graham have transformed their country seat, Easton Park, into a country hospital, and are taking the male surgical cases from the East Suffolk and Ipswich Hospital so soon as they are ready to be moved there to complete their cure. It is not simply a convalescent home, as there has been engaged on the staff three fully qualified and hospital trained nurses or sisters, besides the principals, who are members of the Red Cross Voluntary Aid Detachment, and their co-workers, while a doctor is in constant attendance. By this generous and practical assistance the strain on the hospital's resources is materially relieved, and the many cases of recruits and soldiers which are awaiting operation and treatment can be dealt with much more expeditiously. Some dozen Service men who have undergone major operations at the East Suffolk Hospital are now recuperating at Easton Park, and the generosity of these ladies is not confined to supplying free treatment and maintenance for the patients, but also covers the cost of conveyance from the hospital to Easton Park, and finally to the patients' home or to the nearest railway station. The general exodus of residents from the coast-line inland has had its effect on hospital work in East Anglia—a large number of subscribers have changed their abode, and patients have been brought within the immediate jurisdiction of the hospital who were previously outside its pale. In the absence of a naval engagement of any magnitude the beds of the East Suffolk and Ipswich Hospital have not yet been required for naval wounded, although the hospital's offer of 101 beds for such purpose has been accepted by the Admiralty. The management is now, with the consent of the Admiralty, offering at least a moiety of these beds to the War Office for wounded soldiers, and it is probable a contingent may be sent to Ipswich in the near future, as the first train-load went through to the neighbouring hospital at Norwich this week.

### THE DECLINING VOGUE OF THE OPERATION.

That there are fashions in operations in the classes who can afford to pay for them is not to be denied by those with an intimate knowledge both of surgeons and their patients, and a curious example of its truth has been occasioned by the war. Since its outbreak the number of operations in private practice, nursing homes, and so on appears to have markedly declined, and the suggestion that this is due to the absence of surgeons on active service and to the need for economy among patients cannot be regarded as a wholly satisfactory explanation. For in normal times the greatest sacrifices are made to secure desired or counselled operative treatment among the richer and more prosperous classes alike; and financial prudence is not sufficient to weigh down the scale of the hypochondriac or the man, for such exist, deficient in the sense of the value of his members and organs. On the other hand, though the medical profession is depleted in both its great branches, there are still sufficient operators for private practice, though not enough candidates for house appointments. Yet we are credibly informed that the number of operations which are at all events being postponed is on the increase; that considerably fewer are being demanded; and giving what weight may be due to other causes, the vogue of the operation must be held as partly accountable.

### THE EVOLUTION OF THE HOSPITAL SHIP.

In the course of an interesting article on hospital ships in the *Army and Navy Gazette* the Rev. A. G. Kealy, R.N., has published some interesting researches into the evolution of the hospital ship. He combats the idea that the lately wrecked *Maine* was the first of these sea-going vessels, and quotes 1673 as a date when William Dampier, on the *Royal Prince*, when falling sick before the action of August 11, was sent on board a hospital ship. Other dates are 1692, when Richard Allyn, chaplain of H.M.S. *Centurion*, gives in his list of ships engaged in the battle of La Hogue two hospital ships attached to each Red and Blue Squadron; 1701, when Sir G. Rooke asked for a hospital ship to bring good Thames water to the Fleet, and later ordered "Captain Watkins to take aboard a hospital ship all such sick men as should be sent to him"; 1718, a hospital ship accompanied Sir G. Byng on sailing from Spithead on June 15. Again, in 1793, another chaplain refers to the *Roebuck* as the hospital ship of the Fleet, to which wounded, after the attack in the Isle of Martinique, when the Army and Navy co-operated, were sent. In 1794 a hospital ship was attached to Lord Howe's fleet off Ushant, and in 1795 another to Lord Bridport's fleet off Belle Isle. The writer adds that he can find no record of hospital ships being used in the Crimean War, but that the current *Navy List* contains the names of seven hospital ships carrying nursing sisters, "whose presence at sea," he adds, "is a curious reversal of naval discipline." The presence of nurses with the Army might still be tabooed had it not been for Florence Nightingale; and yet the hospital ship of the present day is not conceivable without women nurses.

### MATERNITY SAFEGUARDS IN WAR-TIME.

The Women's Co-operative Guild, in conjunction with the Women's Labour League and the Railway Women's Guild, representing 40,000 working women, has formed a deputation, which was received by Mr. Herbert Samuel on Monday as President of the Local Government Board and also chairman of the Government Committee for the Prevention and Relief of Distress. The subject of the deputation—namely, the care of maternity in war-time—should be one of the problems to which Mr. Samuel must be fully alive. We have for a long time past devoted a special section of The Hospital to their consideration. The deputation was chiefly concerned with the need for regular and systematic medical advice for expectant and nursing mothers and for children up to school age, and suggested the extension of existing municipal work to maternity centres under the public health authorities. They also proposed that dinners, milk, medical attendance, and midwives could be provided out of payments from the Prince of Wales' Fund. Mr. Samuel, in reply, referred to the provision of more than £1,500,000 in maternity benefit as being already in accordance with the deputation's desires. He suggested that the relief work could be done by the relief committees everywhere established, and remarked that a Government Committee had approved a system of sick-room helps, and that on that day an experiment had started at Plaistow, aided by a grant from the Prince of Wales' Fund. He should rejoice to see the scheme extended, at the cost of the Fund, to women unemployed through the war. He promised finally to send a further communication to all local representative committees emphasising the claims of expectant mothers to relief. On the whole, therefore, the deputation was not turned away empty, thanks probably to the financial aid which the Prince of Wales' Fund has felt within its scope to give.

### HOSPITAL LEGACIES IN TIME OF WAR.

However much the voluntary hospitals in certain centres may be feeling the competition of the various patriotic and relief funds, it is satisfactory to record that legacies so far appear to be coming in satisfactorily. An important instance of this is contained in the will of Miss Margaret Harker Smith, of Tunbridge Wells, who, among other notable gifts to charity, has bequeathed £10,000 to the Homœopathic Hospital of that town, £6,000 to University College Hospital for the Cancer Research Fund, and £5,000 to the New Hospital for Women, Euston Road, London. Subject to private bequests, she left the residue of her large property, which has been sworn at £193,000 odd, to charity. We mention these bequests partly because all such legacies have an added importance at the present crisis, and also in the hope that hospital secretaries and managers, who naturally find that the amount of capital which they receive from this source has a tendency to average itself, will, as soon as sufficient time has elapsed to allow of a valuable estimate, let us know at once to what extent, if any, their legacies, like their subscriptions, are affected by the war.

### A HOSPITAL COMPARED TO BLACK HOLE OF CALCUTTA.

An extraordinarily scathing criticism of the Auchtermuchty District Hospital was given by Colonel Middleton, of Aytoun, at the recent meeting of the Cupar District Committee. After Provost Ferlie had referred in his report to the complaints made against the present hospital by the Local Government Board (who urged the need of a new one), and had moved that certain improvements be carried out, Colonel Middleton seconded. In the course of a speech of the utmost frankness he declared that, while he did not know much about civil hospitals, he had seen many military ones, and he would defy the committee to find in the West of Ireland, in the smallest and dirtiest town, a hospital approaching that at Auchtermuchty. It was not adapted for a hospital, either from its situation or from its structure; its water supply was not as it should be; it was quite unsuited for a hospital, even though "surprising" results, as the Chairman affirmed, were obtained, but failing a better building and a better site the quicker improvements were carried out the better. He did not know how anyone ever came out of it alive, while he knew others who were sent to it who would as soon go to the Black Hole of Calcutta.

### A MEDICAL OFFICER'S RESPONSIBILITY.

Dr. Yule, the county medical officer, said that he agreed with much of what Colonel Middleton had said, but, failing a new hospital, he thought if there were no epidemic, and no sudden increase of population, the present hospital would do if the proposed improvements were carried out. Their cost would be apparently £83. We confess that in our view there are too many "ifs" in Dr. Yule's remarks, and that, as county medical officer, he is not entitled to employ them. If "a great deal" of what Colonel Middleton said is true, as Dr. Yule affirmed, then it is his duty never to permit "the idea of a new hospital" to be "dropped." Surely to countenance in any shape or form conditions remotely resembling the above description is a very grave error of judgment, and one which would recoil most forcibly upon him if his "ifs" happen not to be fulfilled? As county medical officer his principal duty is, and must always be, to maintain the modern up-to-date standard of hospital efficiency in all the public institutions in his area. To do otherwise, to palliate abuses through habit or hopelessness of getting anything done, is no part of his business, and must amount in effect, wherever it occurs, to an indication of failure in the work, which above all else demands pertinacity, obstination, and a relentless standard, in season and out of season, of hospital efficiency.

### CLAIMS OF CIVIL POPULATION AT CARDIFF.

As an instance of how fully the voluntary hospitals are doing their best not to allow the work which they are carrying on for the wounded of the Allied Armies to interfere with that, primarily theirs, for the civil population, we may record the decision of the board of King Edward VII.'s Hospital, Cardiff, to reopen the institution's out-patient department. At a meeting last week the board reported the compliance of the authorities of the 3rd Western General Hospital with their request to hand back the out-patient department for civil use. It was thereupon agreed that the medical members of the special emergency committee should be asked to make arrangements for the reopening of the department, which will probably occur next week. The fact that the hospital's committee requested the military authorities to allow the out-patient department to revert to its former use would appear to indicate how much work there is, which cannot advantageously be postponed, to do in these out-patient departments, even since the Insurance Act came into force. The fact at all events is of much value as indicating that we were not premature in The Hospital of some weeks ago, in respect of the war, to emphasise and insist on the claims of the civil population.

### THIS WEEK'S DRUG MARKET.

The recently noted improvement in the tone of the market is maintained, and, generally speaking, fluctuations in prices are much less violent than they were six weeks or so ago. With some exceptions prices in fact do not appreciably change from week to week. One of the exceptions is carbolic acid, the value of which has continued to advance until at the present time it is three times the price quoted just before the war began, and it seems not improbable that the acid, which is used in warfare in more ways than one, will become still dearer. As carbolic acid is so largely used in hospitals it is important that supplies should be secured on the most economical terms, and as everything points to the probability of higher prices there would appear to be little advantage in delaying the purchase of necessary supplies, notwithstanding the high quotations now ruling. Corrosive sublimate, calomel, and other salts of mercury are also again dearer in consequence of the advance in the price of quicksilver, a metal which is absorbed in large quantities in the manufacture of explosive material. Cod-liver oil has a lower tendency in price, which is remarkable in view of the situation in the North Sea. There is little change in the position of opium. Chamomiles are much dearer; as further shipments from Belgium are not expected, a further advance in price seems probable. Honey is in good supply, as supplies that used to go to Germany are now coming here.

